# Dietary Supplementation With *Tinospora cordifolia* Improves Anxiety-Type Behavior and Cognitive Impairments in Middle-Aged Acyclic Female Rats

**DOI:** 10.3389/fnagi.2022.944144

**Published:** 2022-07-28

**Authors:** Anmol Bhandari, Aditya Sunkaria, Gurcharan Kaur

**Affiliations:** Department of Biotechnology, Guru Nanak Dev University, Amritsar, India

**Keywords:** anxiety, cognitive impairments, memory, menopause, synaptic plasticity, *Tinospora cordifolia*

## Abstract

The midlife transition period in women marks the progressive flattening of neurological health along with increased adiposity, dyslipidemia, frailty, and inflammatory responses mainly attributed to the gradual decline in estrogen levels. Conflicting reports of hormone replacement therapy (HRT) necessitate the exploration of novel therapeutic interventions using bioactive natural products having the least toxicity and a holistic mode of action for the preservation of metabolic homeodynamics with aging in women. The present study was planned to investigate the effects of aging and/or a high-fat diet (HFD) on cognitive impairments and anxiety and further their management by dietary supplement with the *Tinospora cordifolia* stem powder (TCP). Acyclic female rats were included in this study as the model system of the perimenopause phase of women along with young 3–4 months old rats as controls. Rats were fed on with and without TCP supplemented normal chow or HFD for 12 weeks. Animals fed on a TCP supplemented normal chow showed consistent management of body weight over a 12-week regimen although their calorie intake was much higher in comparison to their age-matched controls. Post-regimen, neurobehavioral tests, such as novel object recognition and elevated plus maze, performed on these animals showed improvement in their learning and memory abilities as well as the anxiety-like behavior. Furthermore, due to the presence of multiple components, TCP was observed to modulate the expression of key marker proteins to ameliorate neuroinflammation and apoptosis and promote cell survival and synaptic plasticity in the hippocampus and the prefrontal cortex (PFC) regions of the brain. These findings suggest that TCP supplementation in diet during the midlife transition period in women may be a potential interventional strategy for the management of menopause-associated anxiety and cognitive impairments and healthy aging.

## Introduction

Women passing through a midlife transition, a phase of major neuro-endocrinological alterations, experience impairments in the functioning of multiple organ systems, including the brain (Mosconi et al., 2021). The crosstalk between estrogen decline during the midlife transition and neurological impairments is reported to increase the frailty in post-menopausal women (Schatz et al., 2020). Functions of some cognitive domains such as language/reasoning skills are maintained for the whole lifespan, whereas, frequently, there is an age-associated decline in other cognitive domains related to attention and declarative memory (as reviewed in Gallagher and Rapp, 1997). These functions are under the functional control of the hippocampus and the prefrontal cortex (PFC) regions, and in addition to vulnerability to aging, these functions are also significantly impaired with estrogen deficiency (Keenan et al., 2001). In hippocampi of oophorectomized rats, estrogen was reported to improve synapse development on dendritic spines (McEwen and Alves, 1999; Monk and Brodaty, 2000). In another study, estrogen was shown to increase the choline acetyltransferase activity in the hippocampi and the basal forebrains of oophorectomized rats (Gibbs and Aggarwal, 1998; Markowska and Savonenko, 2002). Along with the aforesaid reports, three meta-analyses projected that women undergoing estrogen treatment after menopause have a 20–40% reduction in the incidence of Alzheimer’s disease (Yaffe et al., 1998; Hogervorst et al., 2000; LeBlanc et al., 2001).

In clinical practice, hormone replacement therapy (HRT) is recommended to manage age-associated neuropathologies in aging women. The conflicting reports of increased risk for breast cancer, pulmonary embolism, and coronary heart diseases with HRT during and/or post-therapy necessitates exploring the safer and more effective alternatives (reviewed in Kargozar et al., 2017). Important criteria while opting for HRT is to consider the “critical window hypothesis,” which means that the age to start the treatment and/or the beginning of the menopause phase determines the effectiveness of HRT, and the benefits are limited to early commencement of the treatment (reviewed in Maki, 2013). In recent times, the demand for complementary therapies and natural products has increased, and aging women prefer to opt for these alternatives to HRT due to their safety, fewer side effects, and presence of phytoestrogens (reviewed in Kargozar et al., 2017).

Globally, there are 34 species belonging to the genus *Tinospora* (Menispermaceae) that are distributed throughout Madagascar, Australia, the Pacific Islands, and tropical Africa (Udayan et al., 2009; Chi et al., 2016). Out of these species, *Tinospora cordifolia*, commonly known as Giloy or Guduchi is a deciduous climbing shrub indigenous to India, known for its vast therapeutic activities from medieval times. It is a high-altitude shrub that bears greenish to yellow flowers (Patil et al., 2021). In the Indian system of medicine, Ayurveda, *T. cordifolia* is commonly known as a miraculous herb having vast pharmacological activities, such as anti-inflammatory (Tiwari et al., 2014), anti-osteoporosis (Gowrishankar et al., 2010), anti-oxidative (Ilaiyaraja and Khanum, 2011), and immunostimulatory (Sengupta et al., 2011). Its estimated annual consumption in India is about 1,000 tonnes (Sharma et al., 2008). All aforesaid activities of this medicinal plant are attributed to the presence of various classes of bioactive molecules, such as alkaloids, sesquiterpenoid, diterpenoid lactones, glycosides, phenolics, and steroids (reviewed in Sharma et al., 2019; Kumar et al., 2020; Sharma et al., 2020a).

Several recent studies from our lab and others have explored the neurotherapeutic potential of this important medicinal plant. Treatment with hydroalcoholic extract of *T. cordifolia* (TCE) induced differentiation and senescence of IMR-32 neuroblastoma and C6 glioblastoma cells (Mishra and Kaur, 2013, 2015). Butanol extract of *T. cordifolia* (B-TCE) and one of its actives, Tinosporicide, ameliorated glutamate-mediated excitotoxic response in the hippocampus, and cerebellar primary neurons (Sharma and Kaur, 2018; Sharma et al., 2020b). TCE and B-TCE were reported to show anxiolytic effects as well as an improvement in cognitive functions in a model of acute-sleep-deprivation rodents (Mishra et al., 2016; Bajaj et al., 2021). Similarly, cognitive function impairments induced by HFD feeding-related obesity in young adult rats were ameliorated with the supplementation of dry stem powder of *T. cordifolia* (TCP) in their diet (Singh et al., 2021). *T. cordifolia* alone or in combination with *Bacopa monnieri* and *Evolvulus alsinoides* was reported to improve the performance of rats in Barnes maze and radial arm maze task performance in scopolamine-induced cognitive dysfunctions (Gupta et al., 2013). In another study, *T. cordifolia* alone or in combination with *Phyllanthus emlica* and fixed-dose formulations with *Bhavana samskara* (combination of both plants coated with the juice of fresh leaves of *Ocimum sanctum*) was reported to improve learning and memory in mice (Malve, 2015).

The majority of studies have used ovariectomized animals as a model system for midlife transition in women. Surgical removal of ovaries disrupts the hormonal reserves causing physiological and behavioral alterations, and it does not mimic the actual biological events associated with a gradual decline in estrogen levels observed in aging women during their midlife transition. Also, it is reported that, in adult rats, ovariectomy induces short-term alterations in the estrogen-targeted structural and functional moieties of neurons, which temporarily affects the emotional behavior (Markowska and Savonenko, 2002; de Chaves et al., 2009). Therefore, middle-aged intact but acyclic female rats were used in this study to mimic the behavioral and functional impairments associated with the midlife transition in transiting women. The current study was aimed to explore the neurotherapeutic potential of TCP as a dietary supplement to ameliorate the age- and HFD-associated neurocognitive impairments and to further identify the underlying molecular targets in the hippocampus and the PFC regions of the brain.

## Materials and Methods

### Procurement and Preparation of *Tinospora cordifolia* Stem Powder

For the preparation of dry TCP, fresh stem samples were procured from the Ropar region of Punjab, India. Stem samples were validated by a plant taxonomist and deposited with the Department of Botanical and Environmental Sciences (Reference no. 65/Bot. and Env. Sci., dated 04/09/2017) at Guru Nanak Dev University, Amritsar, Punjab, India. The stems were fragmented followed by heat drying at 45°C in an oven. Stems were shredded to a fine powder and mixed at a dose of 1 mg of dry stem powder/g body weight of rats with normal chow and HFD for TCP supplementary groups. Dose standardization was done based on our previous lab report, where 1 mg of TCE was calculated to correspond to 6.80 mg of dry stem powder and the reported dose of 140 mg/kg bodyweight was equivalent to 952 mg dry stem powder/kg body weight of rats (Mishra et al., 2016).

### Dosing and Experimental Design

Based on vaginal cytology, Wistar albino female rats aged 12–13 months in the consistent diestrus phase were recruited for this study. To ascertain age-associated cognitive changes in these middle-aged animals, a young control group of 3–4 months old cyclic female rats was also used along with middle-aged animals. Before the experiment, rats were kept in polypropylene cages under controlled temperature and light/dark cycles of 12:12 h with *ad libitum* food and water supply. To investigate the effects of aging and premenopause in middle-aged female acyclic rats, we divided them into five groups (*n* = 5): (1) normal chow-fed young LFD-Y rats (2.5–5% fat, 75–77.5% carbohydrates, 20% proteins as macronutrients); (2) normal chow-fed middle-aged LFD-M rats; (3) TCP supplemented normal chow-fed middle-aged TLFD-M rats (1 mg dry powder/g body weight); (4) HFD fed middle-aged HFD-M rats (30% fat by weight, 50% carbohydrates, 20% proteins as macronutrients); and (5) TCP supplemented HFD fed middle-aged THFD-M rats. Before experimentation, all the animals recruited in this study were fed with *ad libitum* normal chow and water supply. On the first day of experimentation, LFD-Y and LFD-M group rats were continued on normal chow feed; however, TLFD-M group rats were shifted to TCP supplemented normal chow diet, HFD-M group animals were fed 30% HFD, and THFD-M group rats were fed TCP supplemented HFD for the next 12 weeks. Regular feed and calorie intake were estimated per the protocol reported in a previous lab study (Singh et al., 2020). Protocols for all reported experiments were approved (Permission number: 266/2020/12) and conducted as per the institutional policies of “Animal Care and Use” recognized by the Institutional Animal Ethical Committee of Guru Nanak Dev University, Amritsar, India.

### Elevated Plus Maze Test

To assess the age-associated anxiety-like behavior and exploratory activities in middle-aged acyclic female rats, an elevated plus-maze test was conducted on these animals. It is a plus-shaped elevated (50 cm from the ground) apparatus with two opposing open (50 × 10 cm), two opposing closed (50 × 50 × 10 cm) arms, and a central open area. Individual rats from each group (*n* = 7) were placed in the central area, facing toward the open arms, and allowed to explore and move freely for 5 min. The experiment was conducted in a dim-light, clean, and odor- and noise-free room to avoid external disturbance and cues. The movements of each rat, including the number of crossings, the number of entries, the number of head dips, and time spent in the open arm, were video recorded and analyzed (Singh et al., 2021). Anxiety index of animals was calculated in accordance to the formula: Anxiety index = 1 − [(open arm time/5 min) + (open arm entry/total entry)]/2. The obtained values closer to 1 represent a higher extent of anxiety in these animals (Dornellas et al., 2018).

### Novel Object Recognition Test

To assess the effect of age and midlife transition on object recognition and working memory of these middle-aged acyclic female rats, the Novel object recognition (NOR) test was conducted. Rats were habituated to the empty box of dimensions 100 × 50 × 50 cm for 5 min 2–3 days at the start of the dark phase before the main experiment. The activities of rats were video recorded, followed by a familiarization phase during which rats were placed in a box with two similar sample objects without any specific odor and allowed to explore both the objects for 5 min up to 3 days. The sample objects were cleaned thoroughly to ensure the absence of olfactory cues. The least preferred object was replaced with a new object on the day of the test to assess the rats’ recognition memory. Object reconnaissance involved sniffing, licking, chewing, or whisking while the nose was directed at and within 1 cm of the object. Time spent and the number of exploration episodes were recorded and analyzed. The grooming behavior of rats was also tested in the open field box while the NOR test was recorded. Grooming behavior is the time spent by an animal licking various body parts; any grooming bout of more than 5 s was considered an actual grooming bout. Rearing is the behavioral act of standing up on the hind limbs with or without inclining forelimbs on the wall. The number of rearing episodes was also recorded for 5 min sessions during the NOR test. The preference index for the new object was calculated according to the formula: Preference index = Time spent in exploring the new object/Total exploration time (Mishra et al., 2016).

### Western Blotting

After behavior studies, animals from each group were sacrificed, the hippocampus and PFC regions of the brain were dissected, and tissue homogenate in lysis buffer was prepared using a tissue homogenizer at 4°C. The supernatant was prepared by centrifugation of tissue homogenate at 8,000 rpm for 10 min at 4°C, followed by estimation of protein content using Bradford’s method. Preparation of sample and other reagents and sample loading were carried out as per our previous lab study (Singh et al., 2021). Later, the PVDF membrane was probed with rabbit anti-pAkt (1:1,000), rabbit anti-Akt (1:1,000), rabbit anti-AP1 (1:1,000), rabbit anti-BAD (1:1,000), rabbit anti-p-p38MAPK (1:1,000), rabbit anti-BDNF (1:1,000), rabbit anti-TrkB (1:1,500), rabbit anti-p-ERK1/2 (1:1,500) mouse anti-CREB (1:1,000), mouse anti-GFAP (1:5,000), mouse anti-Iba1 (1:1,000) (procured from Sigma-Aldrich, St. Louis, MO, United States); mouse anti-Bcl-xL (1:1,000), rabbit anti-n-NOS (1:1,000) (procured from Cell Signaling Technology, MA, United States); and mouse anti-α-tubulin as internal control (1:5,000) (Sigma-Aldrich, St. Louis, MO, United States) antibodies followed by overnight incubation at 4°C. After overnight incubation, the membrane was normalized at room temperature, followed by three 5-min washes with 0.1% TBST solution. Followed by the three washes, the membrane was then probed with HRP-linked secondary antibodies (anti-mouse and anti-rabbit) and kept at room temperature for 2 h. Visualization and intensity analysis of immunoreactive bands was done per details provided in the previous lab report (Singh et al., 2021).

### Statistical Analysis

Values from five animals in each group (except for behavioral studies, where *n* = 7/group) are expressed as mean ± SEM. The statistical significance of the data was assessed by the one-way analysis of variance (Holm-Sidak *post hoc* method) using the Sigma Stat Software Version 3.5, Systat Software, Inc. A threshold *p*-value of 0.05 was considered to define the statistical significance of different groups reported in this study.

## Results

The current study explored the neurotherapeutic potential of TCP supplementation in amelioratiopn of cognitive impairments and anxiety and to further elucidate the role of protein markers associated with neuroinflammation, cell survival, and synaptic plasticity pathways in the hippocampus and the PFC regions of the brain. For cognitive functioning, neurobehavioral tests, Elevated Plus Maze (EPM) and NOR, were conducted to assess age- and diet-associated changes in anxiety index, memory, and exploratory behavior in these middle-aged acyclic female rats. The current study is in continuation of our previous lab reports, where we reported the active weight management and modulation of metabolic targets of lipid metabolism to ameliorate dyslipidemia by targeting the AMP-activated protein kinase (AMPK) pathway in the liver (Bhandari et al., 2022a).

### *Tinospora cordifolia* Stem Powder Supplementation Ameliorated Anxiety-Like Behavior

**FIGURE 1:**
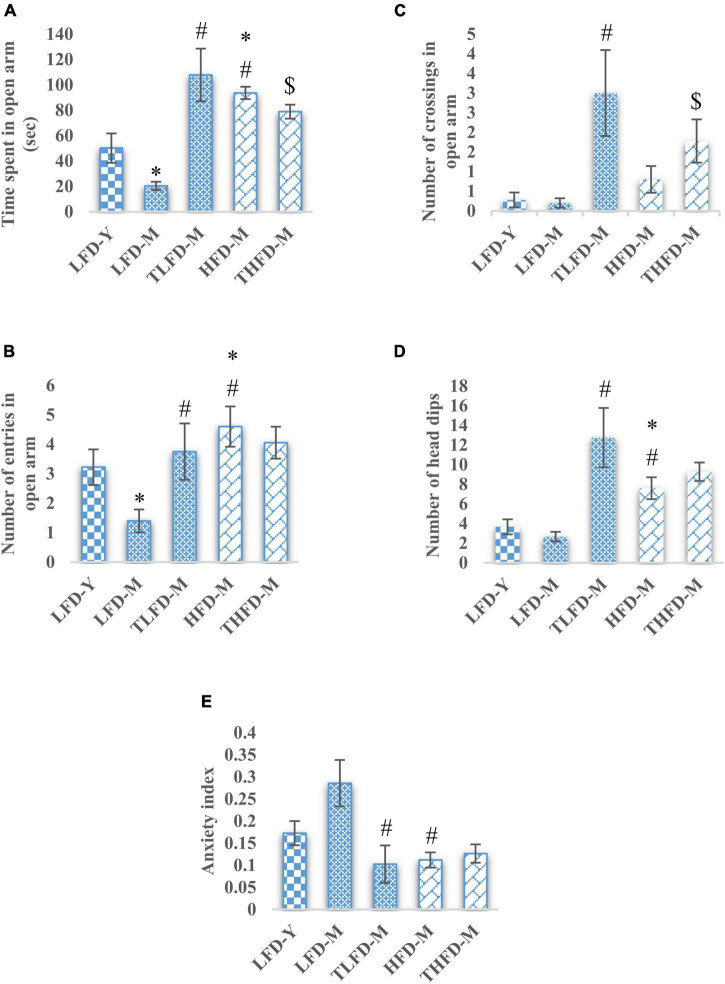
*Tinospora cordifolia* stem powder (TCP) supplementation suppressed the anxiety-like behavior in middle-aged acyclic female rats. Histogram representing **(A)** average time spent in open arm, **(B,C)** number of entries and crossings of rats in open arm, **(D)** number of head dips among different groups of middle-aged acyclic female rats. **(E)** Anxiety index. Values are presented as mean ± SEM. *p ≤ 0.05 LFD-Y vs. LFD-M and HFD-M; #*p* ≤ 0.05 LFD-M vs. HFD-M and TLFD-M; $*p* ≤ 0.05 HFD-M vs. THFD-M.

To assess the anxiety-like behavior in these animals, an EPM test was performed. Normal chow-fed middle-aged LFD-M group rats spent significantly [*F*_(2_,_42)_ = 28.501, *p* < 0.001] less time in the open arm, whereas, HFD-fed middle-aged animals spent significantly [*F*_(2_,_48)_ = 17.448, *p* < 0.001] more time in the open arm compared to young LFD-Y group animals. Among age-matched normal chow and HFD-fed groups, HFD-M group animals spent significantly [*F*_(2_,_45)_ = 150.335, *p* < 0.001] more time in the open arm compared to the LFD-M group. With TCP supplementation in normal chow, TLFD-M group rats spent significantly [*F*_(2_,_42)_ = 28.501, *p* < 0.001] more time, whereas HFD-fed THFD-M group animals spent less [*F*_(2_,_45)_ = 150.335, *p* < 0.001] time in the open arm but more time than the LFD-Y group compared to age- and diet-matched controls (Figure 1A). A similar pattern of changes was observed in the number of crossings and entries in the open arm, which were reduced in LFD-M group rates but increased in HFD-M group rats {significant change in the number of entries in LFD-M [*F*_(2_,_42)_ = 8.842, *p* < 0.001] and HFD-M [*F*_(2_,_48)_ = 3.27, *p* = 0.047]} as compared to young LFD-Y group animals. Moreover, the number of crossings and entries was also higher in HFD-fed HFD-M compared to LFD-M group rats {significant change in the number of entries in LFD-M animals [*F*_(2_,_45)_ = 23.888, *p* < 0.001]}. Further TCP supplementation in the diet was observed to increase the number of crossings and entries in the TLFD-M and THFD-M groups, but a more pronounced increase was observed in the number of entries in TLFD-M [*F*_(2_,_42)_ = 8.842, *p* < 0.001] group animals and in the number of crossings in TLFD-M [*F*_(2_,_42)_ = 22.678, *p* < 0.001] and THFD-M group animals [*F*_(2_,_45)_ = 9.397, *p* < 0.001] (Figures 1B,C).

The anxiety-like behavior of middle-aged LFD-M group animals was also apparent from their less number of head dips, whereas, a significant increase in head dips was observed in HFD-M group rats as compared to both age-matched [*F*_(2_,_45)_ = 29.924, *p* < 0.001] and young animals [*F*_(2_,_48)_ = 18.986, *p* < 0.001]. With TCP supplementation in diet, the number of head dips was increased in animals of middle-age fed on normal chow or HFD; however, the effect was much [*F*_(2_,_42)_ = 29.404, *p* < 0.001] pronounced in normal chow-fed TLFD-M group rats (Figure 1D). Anxiety-like behavior in middle-aged animals was also confirmed by the higher value of anxiety index in normal chow-fed LFD-M group animals compared to young rats, whereas the anxiety index values of TLFD-M [*F*_(2_,_14)_ = 4.72, *p* = 0.031], HFD-M [*F*_(2_,_13)_ = 8.334, *p* = 0.005], and THFD-M group animals were lower than LFD-M but near control LFD-Y group rats (Figure 1E). Overall data suggest that, with aging, LFD-M group animals had higher anxiety scores, which were suppressed with TCP supplementation, whereas HFD showed protective effects against aging.

### *Tinospora cordifolia* Stem Powder Supplementation Enhances the Memory and Exploratory Behavior

**FIGURE 2:**
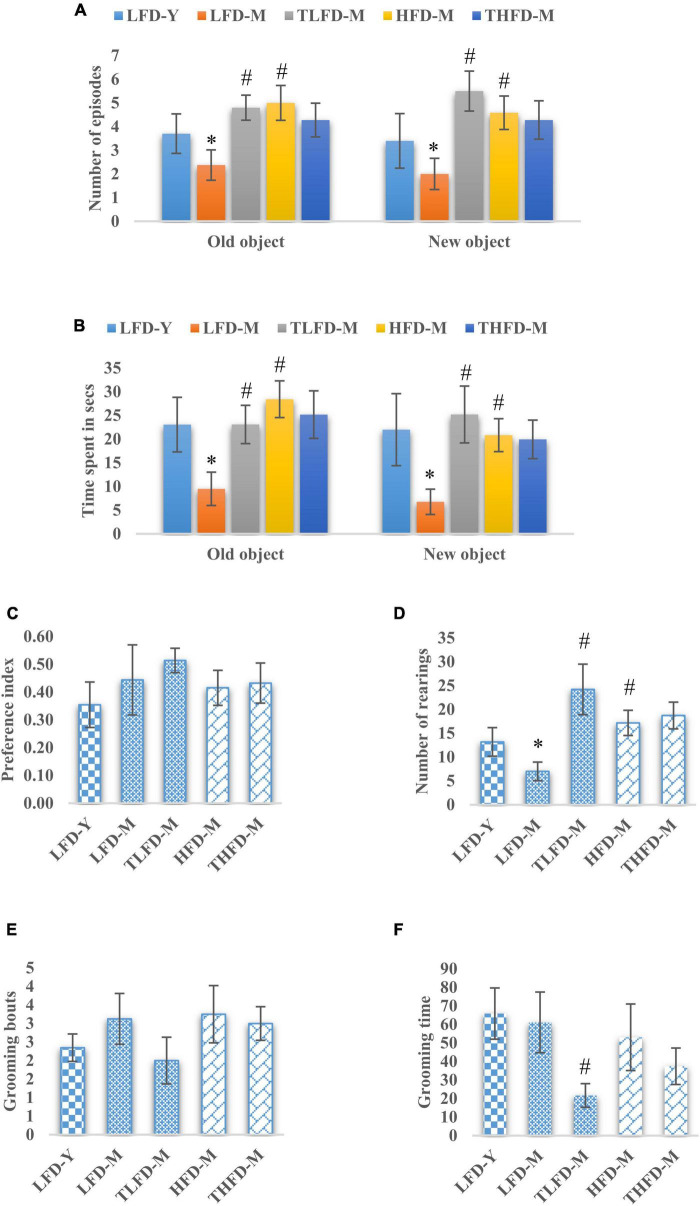
*Tinospora cordifolia* stem powder (TCP) supplementation improved memory and exploratory behavior in middle-aged acyclic female rats. Histogram representing **(A,B)** number of episodes and time spent of the animals, **(C)** preference index of the animals for a new object, **(D)** number of rearing episodes on exploring an object, and **(E,F)** number of grooming bouts and time spent by middle-aged acyclic female rats in grooming activity. Values are presented as mean ± SEM. *p ≤ 0.05 LFD-Y vs. LFD-M; #*p* ≤ 0.05 LFD-M vs. HFD-M and TLFD-M.

To assess memory and learning and exploratory behaviors, the NOR test was performed on these animals. The number of episodes with old and new objects was significantly reduced in LFD-M group animals [old object, *F*_(2_,_33)_ = 12.494, *p* < 0.001 and new object, *F*_(2_,_33)_ = 11.154, *p* < 0.001] but were increased in the HFD-M group as compared to young LFD-Y rats. Age-matched but HFD-fed animals showed a significant increase in the number of episodes with both old [*F*_(2_,_35)_ = 21.297, *p* < 0.001] and new objects [*F*_(2_,_35)_ = 15.032, *p* < 0.001] to LFD-M group animals. TCP supplementation in normal chow significantly increased the number of episodes with both old [*F*_(2_,_33)_ = 12.494, *p* < 0.001] and new objects [*F*_(2_,_33)_ = 11.154, *p* < 0.001], whereas no further change was observed in THFD-M group animals (Figure 2A). Similarly, less time was spent with old [*F*_(2_,_33)_ = 7.722, *p* = 0.002] and new objects [*F*_(2_,_33)_ = 6.546, *p* = 0.004] by LFD-M group rats compared to LFD-Y controls. In age-matched middle-aged animals, the time spent with old [*F*_(2_,_35)_ = 17.724, *p* < 0.001] and new objects [*F*_(2_,_35)_ = 11.445, *p* < 0.001] was higher in HFD-M as compared to normal chow-fed LFD-M group animals. In the TCP supplemented TLFD-M group, animals were seen to spend more time [*F*_(2_,_33)_ = 7.722, *p* = 0.002] with old objects and new objects [*F*_(2_,_33)_ = 6.546, *p* = 0.004] compared to a low-fat diet alone fed age-matched LFD-M animals (Figure 2B). Preference for a new object over an old one was also assessed and values are presented as a preference index. TCP supplemented normal chow-fed TLFD-M group middle-aged animals showed the highest value for their preference for the new object as compared to LFD-Y, LFD-M, HFD-M, and THFD-M groups (Figure 2C).

Rearing behavior that depicts the exploratory behavior was also assessed and the number of rearings was significantly [*F*_(2_,_33)_ = 19.886, *p* < 0.001] reduced in the LFD-M group compared to young LFD-Y group animals. However, their number was increased in HFD-M group rats compared to both LFD-M [*F*_(2_,_35)_ = 23.383, *p* < 0.001] and LFD-Y animals. With TCP supplementation, a significant [*F*_(2_,_33)_ = 19.886, *p* < 0.001] increase in rearing episodes was observed only in TLFD-M group rats (Figure 2D). Anxious animals present with more time spent and the number of bouts in grooming themselves. Middle-aged animals either fed on normal chow or HFD feed showed higher values of grooming bouts compared to young LFD-Y rats, and there was a significant decrease in grooming time in the TLFD-M [*F*_(2_,_33)_ = 6.915, *p* = 0.003] group (Figures 2E,F). These observations suggest that TCP supplementation in a normal diet, as well as an HFD diet, improved memory and exploratory behavior, which also aligns with EPM test data for anxiety-like behavior.

### *Tinospora cordifolia* Stem Powder Supplementation Suppressed the Expression of Reactive Gliosis and Microgliosis

**FIGURE 3:**
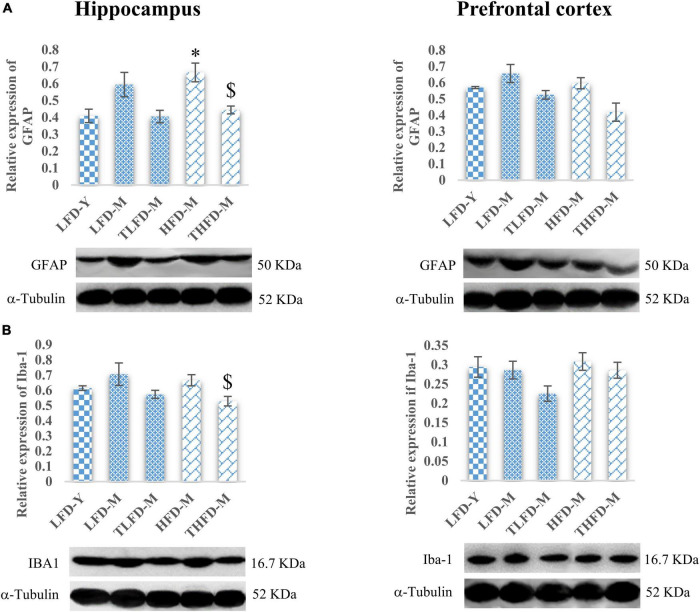
*Tinospora cordifolia* stem powder (TCP) supplementation ameliorated reactive gliosis and microgliosis in middle-aged acyclic female rats. Histogram representing analysis of fold change in expression of **(A)** GFAP and **(B)** IBA1 in the hippocampus (left panel) and the prefrontal cortex (PFC) (right panel) regions of the brain. Values are presented as mean ± SEM. *p ≤ 0.05 LFD-Y vs. HFD-M; $*p* ≤ 0.05 HFD-M vs. THFD-M.

The anxiolytic and memory-enhancing activity of TCP in these female rats was also associated with reduced expression of GFAP and Iba-1 proteins markers of reactive gliosis and microgliosis, respectively, as compared to their age- and diet-matched controls in the hippocampus and the PFC region of the brain. GFAP expression was upregulated with age as observed in middle-aged animals in both the hippocampus and the PFC regions of the brain with a significant [*F*_(2_,_7)_ = 7.755, *p* = 0.017] increase in the hippocampus region of HFD-fed animals as compared to young LFD-Y rats, which were significantly [*F*_(2_,_7)_ = 7.755, *p* = 0.017] reduced with TCP supplementation (Figure 3A). A similar pattern of changes was also observed in the expression of Iba-1 and TCP supplementation, which suppressed these alterations with a significant [*F*_(2_,_10)_ = 4.69, *p* = 0.037] reduction in the hippocampus region of THFD-M animals (Figure 3B). Also, in our previous study, TCP supplementation was observed to suppress the serum levels of pro-inflammatory markers, such as IL-6, IL-1β, MCP-1, and TNF-α, in middle-aged animals fed on either normal chow or HFD feed (Bhandari et al., 2022a).

### *Tinospora cordifolia* Stem Powder Supplementation Modulated the Expression of Apoptosis Proteins and Promoted Cell Survival

**FIGURE 4:**
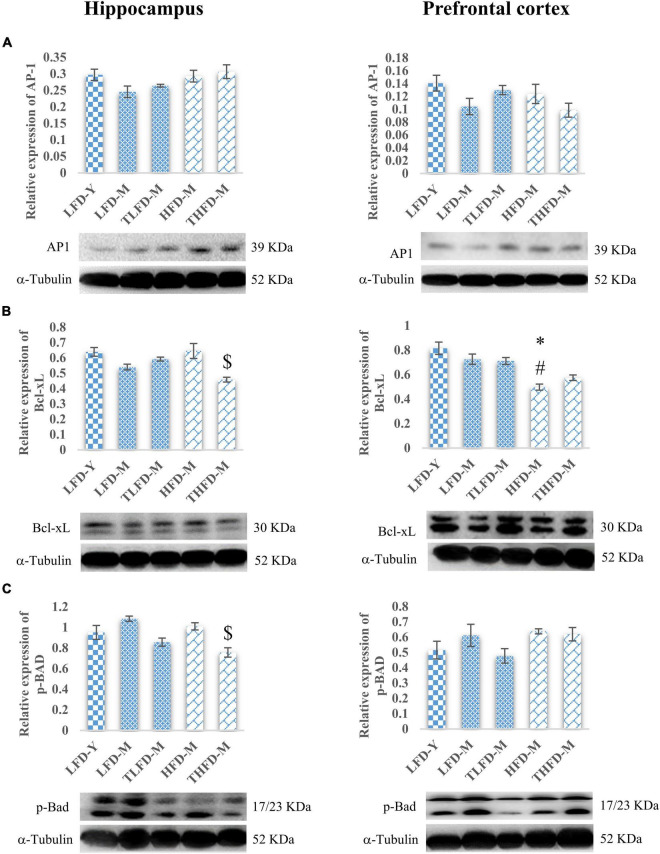
*Tinospora cordifolia* stem powder (TCP) supplementation modulated the expression of target proteins associated with apoptosis in middle-aged acyclic female rats. Histogram representing analysis of fold change in expression of **(A)** AP1, **(B)** Bcl-xL, and **(C)** p-Bad in the hippocampus (left panel) and the prefrontal cortex (PFC) (right panel) regions of the brain. Values are presented as mean ± SEM. *p ≤ 0.05 LFD-Y vs. HFD-M; #*p* ≤ 0.05 LFD-M vs. HFD-M; $*p* ≤ 0.05 HFD-M vs. THFD-M.

Age-associated decline in cell survival and anti-apoptotic proteins, AP-1 and Bcl-xL, was observed in the hippocampus and the PFC regions of the brain in LFD-M group rats as compared to young LFD-Y animals. The expression of AP-1 and Bcl-xL was not altered in the hippocampus region of HFD-fed HFD-M animals; however, in the PFC region, significant [*F*_(2_,_8)_ = 17.295, *p* = 0.001] downregulation in the expression of Bcl-xL was observed in these animals compared to young LFD-Y and age-matched LFD-M group rats. With TCP supplementation, expression of AP-1 and Bcl-xL markers was marginally recovered in TLFD-M group rats compared to age-matched LFD-M group rats, whereas THFD-M rats showed no change in the AP-1 expression and a significant [*F*_(2_,_8)_ = 7.298, *p* = 0.014] decline in the Bcl-xL expression in the hippocampus region. In the PFC region, the Bcl-xL expression was significantly lower in HFD-M than LFD-Y [*F*_(2_,_8)_ = 17.295, *p* = 0.001] and LFD-M [*F*_(2_,_8)_ = 10.338, *p* = 0.017] group animals (Figures 4A,B). On the other hand, pro-apoptotic marker p-BAD showed age-associated upregulation in expression in both LFD-M and HFD-M group rats compared to LFD-Y animals. The expression of p-BAD was similar between age-matched LFD-M and HFD-M group animals, whereas, in TCP supplemented groups, the p-BAD expression was downregulated in TLFD-M and THFD-M group rats in both the hippocampus and the PFC regions of the brain, with a more pronounced [*F*_(2_,_6)_ = 13.443, *p* = 0.006] decrease in the hippocampus region of THFD-M group rats (Figure 4C). These findings suggest that TCP in addition to its anti-inflammatory activity also has the potential to promote cell survival and suppress apoptosis.

### *Tinospora cordifolia* Stem Powder Supplementation Acted Through Synaptic Plasticity and Cell Survival Pathways

**FIGURE 5:**
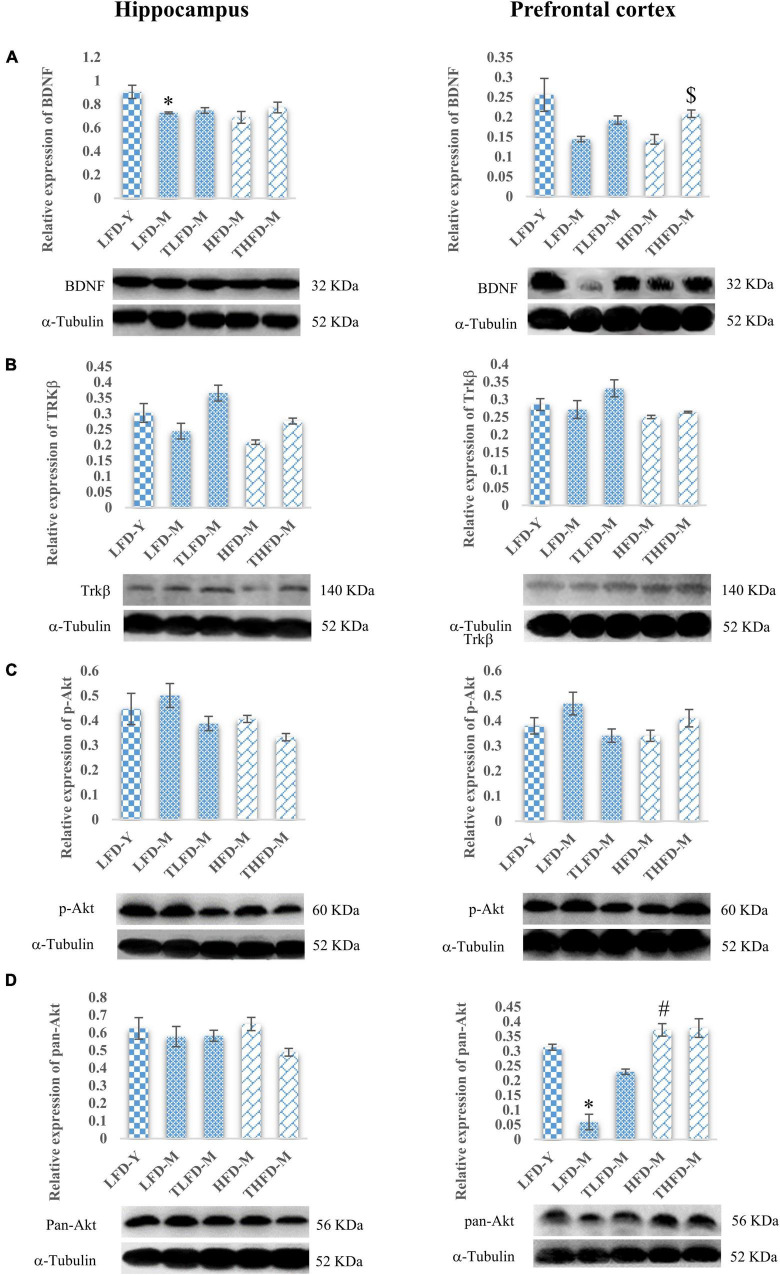
*Tinospora cordifolia* stem powder (TCP) supplementation modulated the expression of target proteins associated with synaptic plasticity and cell survival BDNF/Trkβ pathway in middle-aged acyclic female rats. Histogram representing analysis of fold change in the expression of **(A)** BDNF, **(B)** Trkβ, **(C)** p-Akt, and **(D)** pan-Akt in the hippocampus (left panel) and the prefrontal cortex (PFC) (right panel) regions of the brain. Values are presented as mean ± SEM. *p ≤ 0.05 LFD-Y vs. LFD-M; #*p* ≤ 0.05 LFD-M vs. HFD-M; $*p* ≤ 0.05 HFD-M vs. THFD-M.

Furthermore, to elucidate the role of neurotrophic signaling molecule BDNF and its downstream target proteins of cell survival and plasticity, the effect of TCP supplementation was studied on their expression in the hippocampus and the PFC regions of the brain. Age-associated decline in the expression of BDNF was observed in both the brain regions of LFD-M and HFD-M group rats as compared to young LFD-Y controls, which was more [*F*_(2_,_8)_ = 6.09, *p* = 0.025] pronounced in the hippocampus region of the LFD-M group animals. With TCP supplementation, the expression of BDNF was restored in TLFD-M and THFD-M group rats in the hippocampus and the PFC region, with a significant [*F*_(2_,_8)_ = 11.778, *p* = 0.004] change in the PFC region of THFD-M group animals (Figure 5A). The expression of the downstream marker Trkβ was also downregulated in middle-aged animals of both normal chow- or HFD-fed groups compared to LFD-Y rats. The expression was recovered in TCP supplemented TLFD-M and THFD-M group animals in the hippocampus and the PFC regions with more pronounced recovery in the TLFD-M group (Figure 5B). The expression of p-Akt was upregulated in the LFD-M middle-aged animals as compared to the young controls, whereas it was downregulated in the HFD-fed HFD-M group animals, and the change was suppressed with TCP supplementation in both the hippocampus and the PFC region of the TLFD-M group rats (Figure 5C). With aging and TCP supplementation, no change was observed in the pan-Akt expression in the hippocampus region, whereas the pan-Akt expression in the PFC region was significantly [*F*_(2_,_8)_ = 7.378, *p* = 0.015] decreased in normal chow-fed LFD-M group rats compared to young animals, which was partially recovered in TLFD-M group animals. Between age-matched middle-aged animals, the pan-Akt expression was significantly [*F*_(2_,_8)_ = 6.207, *p* = 0.024] higher in the HFD-M than in the LFD-M group rats (Figure 5D).

**FIGURE 6:**
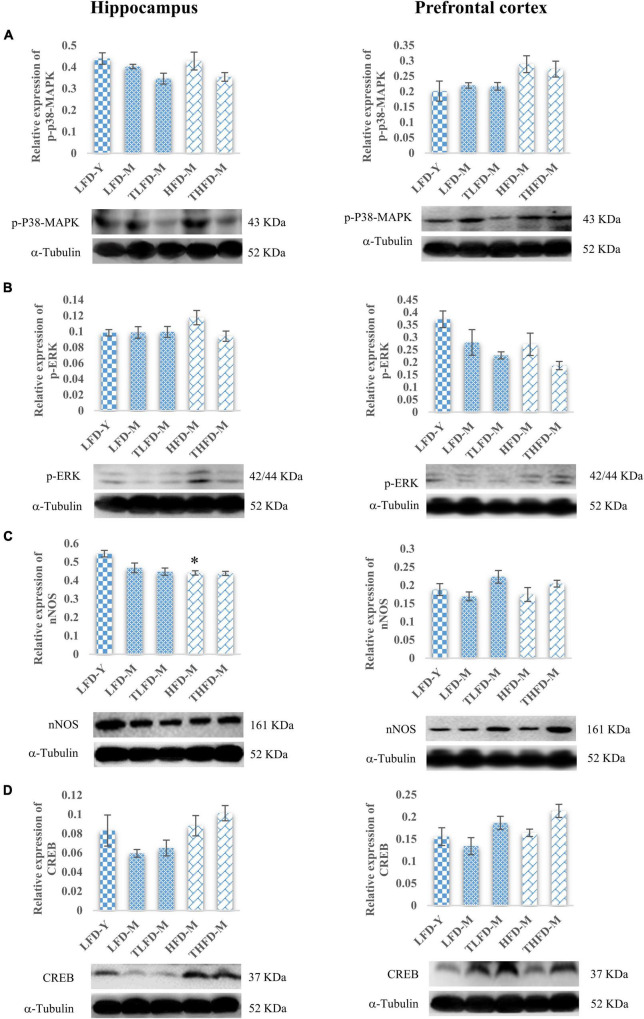
*Tinospora cordifolia* stem powder (TCP) supplementation ameliorated the expression of target proteins associated with synaptic plasticity p38-MAPK/ERK pathway in middle-aged acyclic female rats. Histogram representing analysis of fold change in expression of **(A)** p-P38-MAPK, **(B)** p-ERK, **(C)** nNOS, and **(D)** CREB in the hippocampus (left panel) and the prefrontal cortex (PFC) (right panel) regions of the brain. Values are presented as mean ± SEM. *p ≤ 0.05 LFD-Y vs. HFD-M.

Downstream the BDNF/Trkβ pathway, ERK/MAPK pathway markers were also studied in the hippocampus and the PFC regions. In the hippocampus, no change in the p-p38MAPK expression was observed in normal chow-fed LFD-M and HFD-M group animals with a marginal decrease after TCP supplementation. Similarly, p-p38MAPK expression was not altered in the PFC region in the LFD-M and TLFD-M groups compared to the young LFD-Y group animals. However, the expression of p-p38-MAPK in the PFC region was upregulated in the middle-aged HFD-fed animals, but this change was not statistically significant (Figure 6A). Furthermore, the p-ERK expression in the hippocampus region showed a marginal increase only in HFD-M group rats. On the contrary, in the PFC region, there was downregulation in the expression of p-ERK in LFD-M and HFD-M group animals compared to young control LFD-Y rats. Expression was not restored with different dietary regimens in age-matched middle-aged animals but was reduced in the TLFD-M and THFD-M group animals on the TCP supplemented diet as compared to their age- as well as diet-matched groups (Figure 6B).

Downregulation in the expression of nNOS was observed in middle-aged animals in both the hippocampus and the PFC regions compared to LFD-Y group rats, but the change was statistically significant [*F*_(2_,_7)_ = 12.85, *p* = 0.005] in the HFD-fed HFD-M animals. With TCP supplementation, nNOS was upregulated in the PFC region of both normal chow- or HFD-fed groups (Figure 6C). Furthermore, it was observed that the expression of CREB, a common downstream marker of BDNF/Trk and ERK/MAPK, was also downregulated in middle-aged LFD-M group animals, whereas its expression did not change in HFD-fed HFD-M animals compared to the LFD-Y rats in both the hippocampus and the PFC regions of the brain. With the TCP supplemented diet, the CREB expression was upregulated in the TLFD-M and THFD-M groups, and a more pronounced change was observed in the THFD-M group in the hippocampus and the PFC region of the brain (Figure 6D). The aforementioned data suggest that, due to its multi-component nature, TCP supplementation in diet modulated the expression of molecular targets associated with synaptic plasticity and cell survival pathways in the hippocampus and the PFC regions of the brain.

## Discussion

Middle-aged acyclic female rats (16–17 months old at the time of performing tests) included in the current study were in the persistent diestrus phase of the estrous cycle as evident from their vaginal cytology. Over the dietary regimen period of 12 weeks, all the animals in five groups showed gradual weight gain as compared to their initial body weight (considered to be 100%). Among the normal chow-fed animals, maximum weight gain was observed in the LFD-Y group of young rats in comparison to the middle-aged LFD-M, whereas, TCP supplemented TLFD-M group rats gained minimum weight despite their consumption of almost double the quantity of feed and corresponding calorie intake than their age-matched controls (data published in Bhandari et al., 2022a). On the contrary, feed consumption in rats of HFD-M and THFD-M groups fed on the HFD diet was significantly reduced after about 2 weeks of initiating the regimen, and animals in both these groups had an almost similar feed and calorie intake and their percent bodyweight gain were lower than the LFD-Y, LFD-M, and TLFD-M group rats. Moreover, the dose of TCP used for diet supplementation did not show any toxicity and on the contrary was found to ameliorate aging-associated changes in liver functions and correct dyslipidemia as evident from the data of several metabolic indicators (Bhandari et al., 2022a).

Vulnerability to stress-induced cognitive impairments in women is reported to be higher than that of men (Handa and Weiser, 2014). Age-associated increase in anxiety-like behavior and memory impairments along with lesser exploratory activities was evident from neurobehavioral data of normal chow-fed middle-aged animals. However, TCP supplementation in the diet showed an anxiolytic effect as it was observed to ameliorate the anxiety-like behavior in these middle-aged acyclic animals and also improved their memory and exploratory behavior. Women experience a two times higher risk of developing anxiety disorders, especially in their midlife than men, which are mainly attributed to a decline in the levels of sex hormones in the perimenopause phase (Li and Graham, 2017). Similarly, menstruation, post-partum period, ovarian resection conditions, depression, and anxiety-like behavior are correlated with fluctuating levels of estrogen (Pinkerton et al., 2010). A recent study reported acceleration in anxiety-like behavior in ovariectomized rats on their exposure to additional physical and psychological stressors, which was ameliorated with 17 β-estradiol treatment (Khaleghi et al., 2021). Another study reported that one-year metformin therapy for ovariectomized rats ameliorated the long-term effects of ovariectomy-induced anxiety-like behavior (Zakeri et al., 2019). A previous study from our lab also reported that TCP supplementation ameliorated the obesity-induced anxiety-like behavior in young 3–4 months old female rats (Singh et al., 2021). Similarly, in acute sleep-deprived middle-aged female rats, the B-TCE fraction of *T. cordifolia* was observed to improve the cognitive functioning of middle-aged female rats (Bajaj et al., 2021).

Interestingly, middle-aged animals of the HFD-M and THFD-M groups fed on HFD with and without TCP showed improvement in the aforesaid neurobehavioral tests, thus suggesting that HFD supplementation in middle-aged animals might act as neuroprotective and exerted anxiolytic and memory-enhancing effects. The data are also supported by previous studies showing that palatable food, such as sugar and lard-rich diets, reduced the stress and signs of anxiety in humans and animals (Finger et al., 2011; Singh, 2014). Also, in a recent study, high-fat feeding either enriched with lard or fish oil was observed to possess anxiolytic activity irrespective of their fatty acid composition when fed to young ovariectomized rats (Dornellas et al., 2018). Myristic acid (C16:0) showed anxiolytic activity in male rats, which was comparable to the effects of diazepam (Contreras et al., 2014), and incidentally myristic acid is the most abundant fatty acid in the lard-enriched diet (Dornellas et al., 2015). As compared to normal chow (5% fat) fed young LFD-Y and middle-aged LFD-M group animals, the calories intake was 20 times higher in HFD (30% fat content diet)-fed animals supplemented with or without TCP, but their feed intake was reduced after the first 2 weeks of regimen, and the consumption remained less than normal in chow-fed LFD-Y young and LFD-M middle-aged animals. Therefore, consuming a moderate quantity of lard-based HFD rich in myristic acid and lower gain in their body weight for over 12 weeks may have contributed to their better performance in the EPM and NOR tests.

To further understand the molecular basis of the anxiolytic and memory-enhancing potential of TCP, a detailed mechanistic study of protein markers of neuroinflammation, apoptosis, and synaptic plasticity was done in the hippocampus and the PFC regions of the brain. These two brain regions are known to play an important role in various attention and memory functions (as reviewed in Gallagher and Rapp, 1997) and may serve as important target sites to ameliorate age and estrogen deficiency-driven neurological outcomes. Moreover, the hippocampus region has a major role in the control of mood as well as cognitive functions (Anacker and Hen, 2017). Although this region of the brain is a target of the peripheral endocrine system, its underlying mechanism(s) of action is little explored (Kanoski and Grill, 2017). Interestingly, the hippocampus region is associated with the non-homeostatic regulation of feeding behavior as it regulates reward memory by food-related cues (Davidson et al., 2005). Upregulation in the expression of proteins associated with reactive gliosis (GFAP) and microgliosis (Iba-1) in both the hippocampus and the PFC regions suggests age-associated activation of neuroinflammation, which is further aggravated by the estrogen deficiency state. However, TCP supplementation ameliorated this age-associated upregulation in these protein targets. Peripheral inflammatory molecules activate glial and microglial cells, which, in turn, release pro-inflammatory mediators in the brain tissue. Extended release of these pro-inflammatory markers results in chronic neuroinflammation, thus leading to neurodegeneration (Liu and Hong, 2003).

Also, in elderly people, higher levels of circulatory proinflammatory markers (IL-1β, IL-6, and IL-8) are reported (Singh and Newman, 2011; Ferrucci and Fabbri, 2018). In a parallel study on these animals, we reported age-associated elevation in serum proinflammatory markers (IL-1β, Il-6, MCP-1, and TNF-α) in middle-aged acyclic female rats, and the levels were normalized with TCP supplementation (Bhandari et al., 2022a). Similarly, another recent study reported that the aqueous extract of *T. cordifolia* ameliorated MPTP-induced upregulation in GFAP and Iba-1 expression in the Parkinsonian mouse model system (Birla et al., 2019). Chronic HFD consumption was also shown to increase myeloid cells Iba1+ expression and GFAP+ astrocytes activation in the hypothalamus of the mice brain (Baufeld et al., 2016). Previous studies from our lab also observed that TCP supplementation ameliorated the upregulation in GFAP and Iba-1 expression induced by HFD in the hippocampus and the PFC regions in young rats (Singh et al., 2021) and middle-aged acute-sleep deprived rats (Mishra et al., 2016).

Modulation of anti-/pro-apoptotic markers (AP1, Bcl-xL, and p-BAD) of apoptosis also supports the neurotherapeutic potential of this medicinal plant in middle-aged acyclic females. AP1 is an important protein target to regulate apoptosis in the cells with dual functionality, i.e., its activation is known to promote apoptosis, whereas this protein is also essential for cell survival (Shaulian and Karin, 2001). Bcl-xl is not centered on the regulation of apoptosis, but it governs the protection against mitochondrial damage (Kharbanda et al., 2000), regulation of immune responses (Opferman and Korsmeyer, 2003), the respiration process of the mitochondria (Shimizu et al., 1996), and the repair of DNA (Fan et al., 2000). In addition to these anti-apoptotic targets, the expression of the p-BAD, a pro-apoptotic marker, was also normalized with TCP supplementation. BAD binds with the anti-apoptotic targets of the same family, and the dimerization of BAD with Bcl-xL and Bcl-2, replacing Bas, commences the permeabilization of the mitochondrial membrane leading to apoptosis (Stickles et al., 2015). A transcriptome study from centenarians revealed that the expression of the Bcl-xL gene was upregulated in centenarians compared to septuagenarians and was involved in the regulation of apoptosis, immune responses, and cellular damage control, leading to exceptional aging (Borras et al., 2016). In our previous lab report, TCP supplementation in young adult rats was observed to ameliorate HFD-induced apoptosis in the hippocampus and the PFC regions of the brain (Singh et al., 2021), and one active fraction of *T. cordifolia*, i.e., B-TCE also was effective to ameliorate the acute-sleep deprivation-induced apoptosis in the middle-aged female rats (Bajaj et al., 2021).

To further elucidate the role of neurotrophic factor, BDNF, and its downstream target proteins of the synaptic plasticity pathway, the expression profile of these molecules was explored. TCP supplementation was observed to upregulate the activity of protein targets involved in synaptic plasticity and cell survival in the hippocampus and the PFC region of the aging brain. BDNF shares an important link with brain aging by preventing age-driven cognitive impairments and synaptic loss. Modulation of BDNF and its downstream receptor, Trkβ, is suggestive of the neuroprotective potential of TCP supplementation against age-associated synaptic loss and memory impairments. BDNF signaling *via* synaptic Trk is reported to initiate cellular events that encode memory functions and thus can trigger neurotrophic support (Musumeci and Minichiello, 2011). In another study, DHA was shown to exert learning and memory-enhancing activities owing to increased levels of BDNF in the hippocampus region (Tian et al., 2016). Cellular expression of pan-Akt was downregulated in the PFC region; however, p-Akt was upregulated with aging in middle-aged rats and the change was ameliorated with TCP supplementation, suggesting that TCP supplementation may have enhanced the *de novo* synthesis of pan-Akt in middle-aged rats. Hyperactivation of Akt is associated with many pathological conditions, particularly cancer types (Altomare and Testa, 2005; Bellacosa et al., 2005; Hers et al., 2011). TCP supplementation also modulated the expression of p-p38-MAPK and p-ERK in the hippocampus and the PFC regions of the brain. In a parallel study in our lab to assess the potential beneficial effects of TCP supplementation in diet on motor coordination and cerebellum cell survival and plasticity, we studied the expression of both pan- and phospho-specific forms of p38-MAPK and ERK. As no change in the expression of pan-p38-MAPK and pan-ERK was observed in that study (Bhandari et al., 2022b), we only studied the expression of p-p38-MAPK and p-ERK in this study. However, the expressions of both the markers were high in the hippocampus and the PFC regions of HFD-fed animals, which again suggest the protective role of HFD in aging females. Diet-induced obesity and glucose intolerance were reported to be linked with upregulation in phosphorylation of ERK and p38 markers in mice (MacPherson et al., 2015).

The expression of nNOS, an important neurotransmitter NO synthesizing enzyme, was downregulated in the hippocampus and the PFC regions of both normal chow- and HFD-fed middle-aged animals. Similarly, ovariectomy in a rat model has been shown to downregulate the nNOS expression and subsequent cognitive impairment (Tang et al., 2013). However, this age-associated change was restored with TCP supplementation in these animals, suggesting TCP supplementation as a potential neurotherapeutic agent and improved cognitive functioning in these animals. Expression of CREB, a common downstream marker to BDNF/Trk and ERK/MAPK pathway, was also reduced with aging in middle-aged animals and was alleviated with TCP supplementation. Increased BDNF signaling *via* the Trk-Erk-CREB pathway is reported to inhibit microglial activation and promote neuronal survival in the aging mouse model (Wu et al., 2020). The present data showing that both neurobehavior and neuroinflammation, cell survival, and plasticity marker expression were associated with the midlife transition period in rats agree with the well-characterized homeodynamic role of ovarian hormones (Humeniuk et al., 2011; Kiss et al., 2012; Messina et al., 2013).

## Conclusion

The current data contributed to the identification of key neurochemical and neurobehavioral alterations induced by the gradual decline in ovarian function in acyclic middle-aged rats and the effects of dietary intervention with natural products as well as the positive effects of HFD feeding on the anxiety-type behavior and cognitive impairments. Moreover, the present elucidation of associations between cognitive behavior and underlying regulatory pathways in the brain emphasize the complex relationship between them, and the target proteins may be used as indicators to assess the beneficial effects of such interventions. The neurotherapeutic activity of TCP may be attributed to the presence of its potent bioactive molecules, which alone or synergistically seem to regulate the expression of target proteins associated with these pathways in the hippocampus and the PFC regions of the brain as underlying mechanism(s) to alleviate age-associated cognitive impairments. Although the pre-clinical data is encouraging, before extrapolating the study outcome to humans, we need to work on some limitations of this study, i.e., dose and duration of treatment and post-withdrawal relapses of symptoms (if any). Also, the identification of different bioactive components interacting with protein targets of cell survival and plasticity pathways is important to support the neurotherapeutic claims of *T. cordifolia* to overcome the neurological outcomes in aging women.

## Data Availability Statement

The raw data supporting the conclusions of this article will be made available by the authors, without undue reservation.

## Ethics Statement

The animal study was reviewed and approved by the Institutional Animal Ethical Committee of Guru Nanak Dev University, Amritsar, India.

## Author Contributions

GK obtained the funding. AB conducted the experiments, collected data, and wrote the original manuscript. All authors conceptualized the study, reviewed and edited the final manuscript, contributed to the article, and approved the submitted version.

## Conflict of Interest

The authors declare that the research was conducted in the absence of any commercial or financial relationships that could be construed as a potential conflict of interest.

## Publisher’s Note

All claims expressed in this article are solely those of the authors and do not necessarily represent those of their affiliated organizations, or those of the publisher, the editors and the reviewers. Any product that may be evaluated in this article, or claim that may be made by its manufacturer, is not guaranteed or endorsed by the publisher.
